# Experimental Research of Fibrous Materials for Two-Stage Filtration of the Intake Air of Internal Combustion Engines

**DOI:** 10.3390/ma14237166

**Published:** 2021-11-25

**Authors:** Tadeusz Dziubak, Leszek Bąkała, Sebastian Dominik Dziubak, Kamil Sybilski, Michał Tomaszewski

**Affiliations:** 1 Faculty of Mechanical Engineering, Institute of Vehicles and Transportation, Military University of Technology, 2 gen. Sylwestra Kaliskiego St, 00-908 Warsaw, Polandsebastian.dziubak@wat.edu.pl (S.D.D.); 2 Faculty of Mechanical Engineering, Institute of Mechanics and Computational Engineering, Military University of Technology, 2 gen. Sylwestra Kaliskiego St, 00-908 Warsaw, Poland; kamil.sybilski@wat.edu.pl (K.S.);

**Keywords:** fibrous filter materials, separation efficiency and filtration performance, pressure drop, dust absorption coefficient, two-stage air filter, vehicle combustion engine

## Abstract

Pollutant properties in intake air to internal combustion engines were analyzed. Mineral dust particles’ influence on accelerated engine components’ wear was discussed. Dust concentration values in the air under various operating conditions in trucks and special vehicles were presented. The idea and necessity for using two-stage filters, operating in a “multi-cyclone–porous partition” system for vehicles operated in dusty air conditions, are presented. Information from the literature information has been presented, showing that impurities in small grain sizes reduce fiber bed absorbency. It has been shown that such a phenomenon occurs during filter material operation, located directly behind the inertial filter (multi-cyclone), which off-road vehicles are equipped with. It results in a greater pressure drop intensity increase and a shorter proper filter operation period. It has been shown that filter material selection for the motor vehicle air filter requires knowledge of the mass of stopped dust per filtration unit area (dust absorption coefficient *k_m_*) determined for a given permissible resistance value Δ*p_fdop_*. It has been shown that there is no information on absorption coefficient values for filter materials operating in a two-stage “multi-cyclone–porous partition” separation system. Original methodology and conditions for determining dust absorption coefficient (*k_m_*) of a separation partition, operating under the conditions of two-stage filtration, were presented. The following characteristics were tested: separation efficiency, filtration performance, and pressure drop characteristics of three different filtration partitions. These were A (cellulose), B (cellulose and polyester), and C (cellulose, polyester, and nanofibers layer), working individually and in a two-stage system—behind the cyclone. Granulometric dust composition dosed into the cyclone and cyclone downstream was determined. During tests, conditions corresponding to air filter’s actual operating conditions, including separation speed and dust concentration in the air, were maintained. For the pressure drop values, the dust absorption coefficient (*k_m_*) values of three different filtration partitions (A, B, and C), working individually and in a two-stage system—behind the cyclone—were determined experimentally.

## 1. Introduction

A basic working medium component in every internal combustion engine is air taken from the atmosphere. The amount taken in depends proportionally on engine power. In piston engines, about 700 kW of power is obtained from 1 kg/s (2800 m^3^/h) of air, used to burn fuel. Air flow, sucked in by the internal combustion engine, depends in direct proportion to the engine’s displacement (*V_ss_*), rotational crankshaft speed (*n*), and cylinder filling degree (*η_υ_*)—the values of which depend on engine type (naturally aspirated or supercharged) and charge air cooler presence. As examples of the maximum, theoretically determined air demands for a given engine, see the following: Peugeot 306 Sedan 1.9 D—about 220 m^3^/h; Volkswagen T4 1.9 TDI—about 385 m^3^/h; BWP-1 infantry fighting vehicle—about 1250 m^3^/h; Scania R420 6 × 4—around 1650 m^3^/h; T-72 tank—around 3400 m^3^/h; Leopard 2A1—around 6000 m^3^/h.

Together with air sucked in from the environment, internal combustion engines suck in a significant amount of natural and anthropogenic pollutants. A main component of these pollutants is dust, including mineral dust, called road dust. SiO_2_ silica and Al_2_O_3_ corundum are two basic minerals whose content in dust ranges from 60 to 95%, depending on type and substrate condition. In smaller amounts, dust contains Fe_2_O_3,_ MgO, and CaO. Moreover, in dust, in very small amounts, there are K_2_O, Na_2_O, and SO_3_ [1,2,3].

Silica and corundum, whose content in dust is the highest, also have the highest hardness, at 7 and 9, respectively, on the 10-degree Mohs scale. In addition, dust grains are very irregularly shaped. These are solids with sharp edges. After getting into the engine cylinders with the air, mineral dust penetrates between cylinder and piston frictional surfaces as well as piston rings. As a result of this phenomenon, there is an increased wear to engine elements. The cylinder liner (in its upper part), the piston, and the piston sealing ring are most exposed to abrasive wear. Abrasive wear to engine elements is intensified by mineral particles with sizes in the range 1–40 µm. However, most aggressive are dust particles, whose size does not exceed 20 µm [3,4,5,6]. Excessive wear to the cylinder liner, piston, and rings causes a drop in the piston space tightness, which results in a drop in compression pressure and engine power.

The greatest surface wear to two cooperating elements is caused by dust particles with diameters (*d_z_*) of equivalent size as the oil film thickness (*h_min_*) between two surfaces at a given moment. Oil film thickness, in connections lubricated with engine oil, depends on operating engine conditions (load, crankshaft rotational speed), temperature, and oil viscosity. For this reason, oil film assumes various values, most often in the range of *h_min_* = 0–10 µm [7,8,9]. The authors of [5] state that over 30% of the pollutants that flow into the engine cylinders with the inlet air then flow out, together with exhaust gases. Thus, this dust percentage is not involved in engine components wear but does increase particulate matter emission (PM). Less than 10–20% of dust that is transported with engine intake air settles on the oil film on the cylinder liner wall. Together with oil, this percentage of dust forms a mixture that is dangerous for the piston–rings–cylinder (P–P–C) combination, intensifying abrasive engine components’ wear. The remaining part of the dust is evaporated or oxidized under high pressure and high temperature conditions, occurring during the fuel combustion process in the cylinder [10].

It follows from the above that all mineral dust grains above 1 µm cause accelerated wear; therefore, motor vehicle engine air intake filters must remove them with the greatest possible efficiency.

The dust mass that enters an engine with the intake air depends on the dust concentration in the air around the moving vehicle, which can have a wide variety of values. Typical dust concentrations in the air may vary from 0.01 mg/m^3^ in clean rural environments to about 20 g/m^3^ in desert conditions (during tracked vehicles’ movement) [4,11]. According to the author of [12], dust concentration in dusty air may be in the range of 0.001–10 g/m^3^. The authors of [13] showed during their research that the maximum dust concentration in the air within a few meters of a sandy road, along which all-terrain vehicles traveled, was variable in the range of 0.05–10 g/m^3^. The authors of [14] reported that dust concentration on motorways has low values in the wide range of 0.0004–0.1 g/m^3^, and when driving among a vehicles column on sandy terrain, this reaches 0.03–8 g/m^3^. 

The author of [15], examining dust concentration in the air at a distance of several meters behind a moving trucks column, an armored personnel carriers column, and a column of tracked vehicles, showed that behind the tanks column, dust concentration reached maximum value of 1.17 g/m^3^. On the other hand, the authors of [16] stated that dust concentration measured in close proximity (about 80 mm) to the armor surface of a tracked vehicle, used on sandy terrain, increased with an increase in driving speed—for 18 km/h it ranged from 2.1–3.8 g/m^3^. At the inlet to the air filter, the concentration was much lower, at 0.8–1 g/m^3^.

During the take-off or landing of a helicopter on an accidental landing site, dust concentration in the air at the height of the tip of the CH-53 helicopter propeller (distance above the ground—0.5 m) may reach the value of s = 3.33 g/m^3^ [17]. According to the authors of [4,12,13], the actual dust concentration at the inlet of the inlet system in an internal combustion engine of a vehicle does not usually exceed the value of 2.5 g/m^3^, which is still a very high value. According to the authors of [18], dust concentration in the air in the range of 0.6–0.7 g/m^3^ significantly reduces visibility, and at a concentration of about 1.5 g/m^3^, the visibility is zero.

Large dust amounts in the air come from friction elements, such as brakes wear, car tires, and road surfaces [19]. The research presented in [20] showed that dust emission from road surface wear, tire tread and friction elements of car systems (emissions other than exhaust emissions) exceeded the emission of pollutants from car exhaust gases. During the braking process, as a result of friction between the brake pad and the brake disc, a large amount of thermal energy is released, and surfaces are abraded, producing brake dust consisting mainly of heavy metals. The authors of [21] determined that brake dust composition is mainly made up of S, Ca, Fe, Cu, Ba, and other elements, most of which are metals. Brake dust particle size (TSP) does not exceed 50 µm. It was found that the finest dust particles (below 2.5 µm) account for approximately 42% of brake dust, and dust particles with a size of 2.5–10 µm account for approximately 40%. Therefore, brake dust is an important component of PM 2.5 and PM 10 pollutants.

Large dust amounts are generated during coal open-cast mines operation [22,23,24]: a place where trucks and working machines are used. They are equipped with high-power engines, and therefore, have a large air requirement, sucking in a large amount of dust. The authors of [23] analyzed dust distribution in a Chinese open-cast mine and found a very high (426 g/m^3^) dust concentration around the working area of the devices. On the other hand, the authors of [24] determined dust concentrations in the Pingshuo mining area in China at about 60 g/m^3^, 114 g/m^3^, and 250 g/m^3^ for PM2.5, PM10, and TSP, respectively.

It follows from the above that trucks, special vehicles (tanks, armored personnel carriers, and infantry fighting vehicles), and working machines are used in conditions with high dust concentration values in the air. These vehicles’ engines draw in a considerable dust amount with the air. For example, the engine of a PT-91 tracked vehicle with a capacity of 625 kW draws in more than 3400 m^3^/h of atmospheric air per hour. If dust concentration in the air is 1.5 g/m^3^, a motor draws in more than 5 kg of dust, with the air, per hour.

The basic filtering materials for engine inlet air are filter papers with a high separation efficiency—over 99.5%. However, the pressure drop of these filter types tends to increase rapidly when they operate in high dust concentration conditions, necessitating frequent, periodic filter element replacements. Therefore, in order to remove large dust mass in a short time and reduce the dust load of high-efficiency filters—extending their service life and reduce operating costs—two-stage filtration systems are used, consisting of a pre-filter that removes large particles (above 25–35 µm) and main filter to trap finer particles (above 2–5 µm). Two-stage air filters differ in design, principle, and operation of the first filtration step, filter partition types, and operation effectiveness.

**Figure 1:**
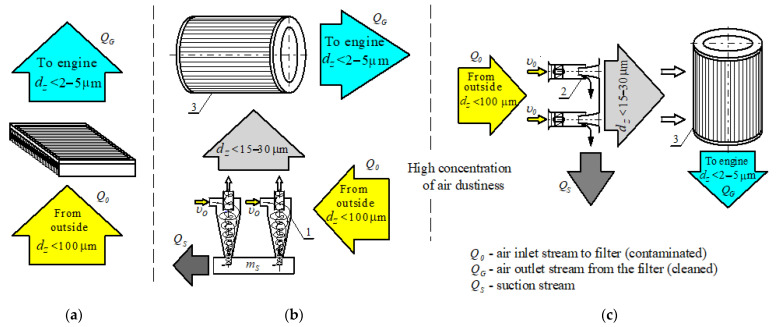
Air filtration system in the filter: (**a**) single-stage (porous barrier); (**b**) two-stage (multi-cyclone of return cyclones with a tangential inlet–porous barrier); (**c**) two-stage (multi-cyclone of axial plow cyclones–porous barrier).

The first air filtration stage (pre-stage filter) is generally a multi-cyclone, which is a set of individual cyclones with internal diameters not exceeding *D* = 40 mm, arranged in parallel (next to each other). They are fixed, with their ends in the common lower and upper plates, which guarantees a common air inlet and outlet. Multicyclones can be constructed of tangential inlet reflux cyclones or flow cyclones (Figure 1).

The second air filtration stage is a partition element (filter cartridge), which, due to space limitations and the desire to increase the surface area of the filter material, is most often made of pleated filter paper [25].

A two-stage filter can be a set of two baffle filters (initial and main), arranged in series, with an appropriately selected structure [26]. In this case, the efficiency of the pre-filter is significantly influenced by particle size distribution. Pre-filter protection is more pronounced with large particles and tends to disappear as the proportion of fine particles increases.

Installing a pre-filter, in the form of an inertial or baffle filter, upstream of the main filter, has many advantages, especially when the aerosol is dominated by coarse particles. It can greatly reduce the pressure drop’s increase in intensity on the main filter, as well as effectively increasing total particle retention capacity of the entire separation system, and thus, extends the main filter’s working time until the permissible resistance is reached.

Depending on the conditions during vehicle movement, air sucked in by the filter intake contains dust grains with dimensions not exceeding *d_p_* = 80 µm. The essence of the operation of a two-stage filter “multi-cyclone–porous partition” is that a multi-cyclone is characterized by the possibility of separating large dust masses from polluted air without increasing the pressure drop, but with a not very high efficiency (87–95%) or filtration performance (*d_p_* > 15–35 µm). Remaining insignificant dust mass is directed to filter element, most often made of pleated filter material (most often paper) with low and limited absorbency (*k_m_* = 200 g/m^2^), but a high filtration performance—above *d_p_* = 2–5 µm—and high separation efficiency—above *φ* = 99.5% [27,28,29,30].

On the other hand, fibrous materials from nanofibers, for example PA-56 membrane, consisting of completely ultra-thin (20 nm) nano-nets, show a high separation efficiency of 99.995% and a low pressure drop of 111 Pa, combined with a high dust holding capacity of 49 g/m^2^ [30].

An absorption filter material’s capacity is characterized by the dust mass loading coefficient (*k_m_*). It is quotient of dust mass (*m_cw_*), which has been retained and evenly distributed on the surface of the tested filter material, until the filter reaches the agreed permissible resistance value (Δ*p_fdop_*) and active filter paper surface (*A_c_*):

(1) $$k_{m}=\frac{m_{cw}}{A_{c}}\;[g/m^{2}]$$

Dust absorption coefficient values (*k_m_*) for standard filter materials made of cellulose were determined during experimental work, using dust with grain sizes below 80 µm, and they have values in the range *k_m_* = 190–240 g/m^2^ [31,32,33]. Dust with this grain size (standard dust) is directed directly with the air onto the filter element of single-stage filter.

For intake air filtration in modern motor vehicle engines, other materials are also used, which are composite layers, for example: polyester, cellulose, and nanofibers. These materials show much greater efficiency and filtration accuracy, but they achieve lower dust absorption coefficient values (*k_m_*). The authors of [34], while examining composite filter materials, obtained—with flow resistance Δ*p* = 3 kPa—different dust absorption coefficient values, respectively, as follows: polyester—*k_m_* = 135 g/m^2^; cellulose with a polyester layer—*k_m_* = 120 g/m^2^; cellulose with polyester and nanofibers layers—*k_m_* = 102 g/m^2^.

The authors of [35] examined a filter material consisting of two layers and found that dust absorption coefficient values are influenced by the inlet layer type. When there was a microfiber layer at the inlet, the two-layer filter bed obtained—with a pressure drop of 2 kPa—the absorption coefficient *k_m_* = 84 g/m^2^. On the other hand, when a sub-microfiber filter layer was placed at the inlet, the obtained coefficient value (*k_m_* = 95 g/m^2^) was much higher (by 13%).

For non-woven filters used as a filtering medium for air filters in passenger car engines, the dust absorption coefficient (*k_m_*) obtains much higher values (*k_m_* = 350–500 g/m^2^) than the filter paper. This is mainly due to much greater thickness of the non-woven filters (2–5 mm) than the filter paper, the thickness of which does not exceed 1 mm. The authors of [36] state that the absorbency coefficient values of calendared non-woven fabrics with a thickness of 3.2 mm, obtained during their research, were *k_m_* = 85.5–112.3 g/m^2^. For the same fleece, but not calendared, with the same flow resistance of 0.3 kPa, the absorbency coefficient had the value of *k_m_* = 54.5–89.3 g/m^2^, which was much lower.

According to the authors of [37], who experimentally tested high-performance glass fiber filter media (thickness of 0.33 mm and grammage of 93.4 g/m^2^), they showed that the dust absorption coefficient of the bed at the moment of reaching pressure drop was 2.5 kPa, at the separation speed of 0.1 m/s and dust concentration 0.32–7.08 µg/cm^3^, with values in the range 125–220 g/m^2^.

As a result of two-stage filtration application, air is supplied to the engine cylinders with the required purity and, at the same time, the filtration system’s operation time is extended, and thus the vehicle service interval—limited by reaching a certain value of pressure drop—reaches the permissible resistance (Δ*p_fdop_*). Values of permissible resistance are determined for passenger car engine air filters by using the criterion of a 3% decrease in engine power and assuming values in range of 2.5–4.0 kPa. For truck engines, permissible resistance is then 4–7 kPa [4], and for special purpose vehicles it is Δ*p_fdop_* = 9–12 kPa [38].

Filter materials for internal combustion engines’ intake of air are usually made of cellulose as it is cheap and easy to process. However, cellulose media are characterized by low initial efficiency and filtration accuracy, which may be affected by accelerated engine wear and durability, and a relatively large pressure drop resulting from dust accumulation on filter bed. These problems have been partially solved by new filter media structures use that are a composite of several layers: synthetic nanofibers and standard cellulose filter media. Most often, a layer of nanofibers 1–5 µm thick and fiber diameters 300–800 nm is applied to a standard filter bed made of fibers with a diameter of 10–15 µm [39,40,41,42,43,44], which improves dust grains separation efficiency below 5 µm in the air sucked into the engine. This significantly increases engine durability, but also more intensively increases filter pressure drop.

Filter paper is shaped in the form of a pleated tape, from which filter inserts are then made. At the same time, a filter element should be shaped to obtain the maximum active filter surface of the paper in a given volume. At the same time, maximum filtration speed criterion (*υ_Fmax_*) should be kept, which assumes that, for passenger car filters, the value of 0.07–0.12 m/s should not be exceeded, and for truck filters 0.03–0.06 m/s should not be exceeded [45,46,47,48,49].

Filtration speed is conventional average speed, defined as quotient of air stream flowing through the filter cartridge and filter paper active surfaces. Maximum filtration speed (*υ_Fmax_*) is determined from the following:

(2) $$\upsilon_{Fmax}=\frac{Q_{Fmax}}{A_{c}\cdot 3600}\;[m/s],$$

where *Q_Fmax_* indicates the nominal air flow that flows through the filter element, equal to engine air demand (*Q_Silmax_*) (m^3^/h) at the maximum power rotational speed and 100% mixture throttle opening (engines with SI); *A_c_*—active filter paper cartridge surface [m^2^].

The maximum filtration speed (*υ_Fmax_*) is the basic criterion used when selecting active surface (*A_c_*) filter paper for car engine air filters. Experience has shown that this factor is not sufficient to ensure required air filter operating time, and thus vehicle mileage. The significant air filter working time limitation is achieved by the filter flow resistance permissible value (Δ*p_fdop_*)—this being the criterion for the end of its life. Filter operation time to obtain the Δ*p_fdop_* value depends not only on filtration speed (air stream) in porous partition, but also on its absorption, which directly depends on filter material structure and dust grain sizes.

A single-stage filter, the filtering element of which is most often a pleated filter paper cartridge, along with air, sucks up dust grains directly from the environment, with dimensions not exceeding the value of *d_p_* = 80 µm. In a two-stage air separation system (multi-cyclone–filter paper) in truck engines and special vehicles, “after passing” through the cyclones, dust has a substantially different fractional composition; then, dust grains of much smaller sizes (not exceeding *d_p_* = 15–35 µm) are transferred to the second separation stage (paper filter) [50,51,52,53,54,55,56]. Therefore, the separation process, in cases where the paper filter is second (after the multi-cyclone), the separation stage may be completely different than that of a single-stage filter. When small-sized dust flows with air onto a porous barrier (filter paper, non-woven fabric), the pressure drop increases more rapidly as a result of lower air permeability through the formed dust layer. Consequently, the filter reaches the Δ*p_fdop_* value much earlier, with a smaller retained dust mass. Such a phenomenon was observed during the experiments in [57,58,59,60,61] and numerical [62,63] fibrous material tests as well as in “cyclone–pleated filter paper” unit experimental tests [64,65].

For example, the authors of [57] investigated a non-woven bed using two dusts with different particle distributions. Dust with a particle size of 0–12 µm caused the fastest increase in pressure drop (*k_m_* = 400 g/m^2^) and for loading it obtained the value of 7 kPa. Dust with larger particle sizes of 0–20 µm caused a lower intensity increase in pressure drop of the tested bed, hence the value of 7 kPa was obtained for the larger value of *k_m_* = 800 g/m^2^. A similar phenomenon was observed during tests of a synthetic bed, with three dusts with different granulometric compositions: dust with a size of 0–10 µm, AC fine (0–80 µm), and AC Coarse (0–200 µm) [58]. The smaller the size of the dust grains, the more intense the increase in the pressure drop. At a separation speed of *υ_F_* = 0.05 m/s and a determined pressure drop value of 0.25 kPa, bed loading with dust was *k_m_* = 125 g/m^2^, *k_m_* = 165 g/m^2^ and *k_m_* = 360 g/m^2^, respectively. For greater separation velocity (*υ_F_*) values, bed dust loading—for a predetermined pressure drop value—assumes, irrespective of dust type, proportionally lower and lower values due to a more intensive increase in pressure drop.

In [59], changes in the cellulose pressure drop of the filter bed, depending on dust loading, were investigated. Four dusts with a similar chemical composition but with different granulometric compositions were used: No. 1, 0–10 µm; No. 2, 0–20 µm; No. 3, 0–40 µm; No. 4, AC Coarse, 0–200 µm. Fine dust increases the pressure drop more intensively, which results in lower dust mass retained per unit area. For the same pressure drop value (Δ*p_f_* = 5 kPa), loading the filter bed with dust takes the following values: *k_m_*_1_ = 75 g/m^2^, *k_m_*_2_ = 210 g/m^2^, *k_m_*_3_ = 310 g/m^2^, and *k_m_*_4_ = 560 g/m^2^.

It was shown in [60] that a filter bed loaded with 0.46 µm particles shows a faster pressure drop than that with 1.40 µm particles [60]. For an assumed pressure drop value of 2.5 kPa, the dust load for 0.46 µm particles is *k_m_* = 32 g/m^2^, and for 1.40 µm particles it is *k_m_* = 53 g/m^2^, which is almost twice as much.

The authors of [61] presented pressure drop modeling during surface separation of PTFE HEPA filter material for applications with low dust load. Experimental pressure drop curves were obtained using monodisperse SiO_2_ dust with different diameters, as follows: *d_p_* = 52, 103, 310, and 553 nm. Fine dusts caused a more intensive increase in bed pressure drop than coarse dusts. Dust grains with the size *d_p_* = 52 nm cause the bed to achieve a pressure drop of Δ*p_f_* = 0.6 kPa when loaded with dust, *k_m_* = 1.2 g/m^2^. With the increase in dust grains size, bed loading with dust increases for smaller and smaller pressure drop (Δ*p_f_*) values. The bed into which dust size *d_p_* = 553 nm was dosed, obtained a dust load of *k_m_* = 5.5 g/m^2^ with a pressure drop of Δ*p_f_* = 2.5 kPa.

The authors of [62] tested several types of filter media, containing cellulose, synthetic (felt), glass, double-layer glass/cellulose, and mixed synthetic/glass. Flow effectiveness and resistance of selected deposits, depending on dust load, were investigated. Two test dusts were used: SAE Fine and submicron alumina powder (median diameter 0.25 μm), containing 99% Al(OH)_3_. Results showed that dust load depends on particle size distribution and filter type. Submicron particles cause filter beds pressure drop to increase much faster than when SAE Fine dust particles are operating. Moreover, cellulose filters with a layer of very fine fibers on the surface showed the fastest increase in pressure drop due to dust loading.

The authors of [63] examined pressure drop on industrial filters exposed to fine alumina (median diameter 0.25 μm), containing 99% Al(OH)_3_, and coarse Arizona dust with the chemical composition of SiO_2_ (65–75%), Al_2_O_3_ (11–17%), Fe_2_O_3_ (2.5–5%), Na_2_O (2–4%), CaO (2–5%), MgO (1–2%), and TiO_2_ (0.5–1%). They found that pressure drop was strongly dependent on the ratio of fine to coarse particles.

The greater the mass ratio of fine particles to coarse particles, the faster the pressure drop increased. If filter bed received only fine dust, a pressure drop of 850 kPa was obtained for dust loading *k_m_* = 1.5 mg/cm^2^ (*k_m_* = 15 g/m^2^). Such a pressure drop value on the filter bed for Arizona dust (coarse) was obtained for a much higher dust load *k_m_* = 15 mg/cm^2^ (*k_m_* = 150 g/m^2^). 

The authors of [64] conducted experimental tests of a filter cartridge with AC-301 pleated non-woven fabric with a thickness of *g* = 2.5 mm, operating in the conditions of one-stage and two-stage filtration, using the multicyclone of axial flow cyclones.

Polydisperse dust with particle size *d_pmax_* = 80 µm was used. A permissible pressure drop value Δ*p_w_* = 5 kPa was achieved by the non-woven filter element operating in the conditions of single-stage filtration with dust absorption coefficient *k_m1_* = 700 g/m^2^, i.e., with a value twice as high as in the conditions of two-stage filtration—*k_m2_* = 325 g/m^2^ [64]. Thus, the absorptive filter fabric capacity, on which the dust flows—the particle size composition of which has been changed in the inertial filter (*d_p_* < 20–35 µm)—is 50% lower than that of the non-woven fabric on which dust flows directly from the environment (*d_p_* < 80 µm).

The authors of [65] conducted experimental tests of a cylindrical filter cartridge made of pleated filter paper with a thickness of *g* = 0.56 mm, operating in the conditions of one-stage and two-stage filtration. A single axial flow cyclone was used as the first separation stage. Polydisperse dust with particle size *d_pmax_* = 80 µm was used. Working in the conditions of single-stage separation, dust absorption coefficient of the tested filter paper, with the resistance value of Δ*p_w_* = 5 kPa, reaches following values: *k_m_* = 278 g/m^2^ for standard dust and *k_m_* = 210 g/m^2^ when the paper element worked as the second filtration stage.

The above data shows that the dust absorption of the barrier for small grains is much lower than when the barrier is loaded with large-grained dust particles. When small-sized dust flows with the air onto the porous barrier (filter paper, non-woven fabric), grains retained on the fibers form expanding dendrites that fills free spaces (pores) between the fibers, creating a more compact and thus less permeable structure. Pressure drop increases more rapidly as a result of lower air permeability through the dust layer. Therefore, the filter obtains a Δ*p_f_* value much earlier and with a smaller retained dust mass per unit of filtration area—lower dust absorption coefficient value (*k_m_*). This phenomenon occurs during operation of two-stage air filter “multi-cyclone–paper partition”, where large-size dust grains are retained by the multi-cyclone, and small ones are directed to filtration partition. Therefore, for the proper design of a two-stage air filter, it is necessary to know the dust absorption coefficient *k_m_* of the filter material, determined in the conditions of two-stage filtration. Coefficient *k_m_* value is particularly important when forecasting the distance traveled by the vehicle until the filter reaches the permissible flow resistance value Δ*p_fdop_*. Filter material producers do not provide such data, only providing the following paper structure parameters: pore size, air permeability, and thickness. There is no data on filtration properties with dust use, in particular, filter bed dust absorption, i.e., dust mass per material unit area. Such data can be obtained during numerical tests as well as experimental material samples. Experimental research is costly and labor intensive; however, it is the most reliable research method. The problems of small-grain dust separation in a pleated (non-woven) paper filter, working directly downstream of an inertia filter, is not sufficiently researched or described in the available literature. In order to fill the gap in this respect, this paper presents an experimental determination methodology of the dust absorption coefficient of any filter material operating in a two-stage system—directly after the inertial filter. The methodology consists in examining the separation characteristics of any material sample, working directly downstream of a single cyclone in laboratory conditions, while maintaining the flow conditions of an actual air filter.

## 2. Own Experimental Research

### 2.1. Purpose, Scope, and Research Subject

**Figure 2:**
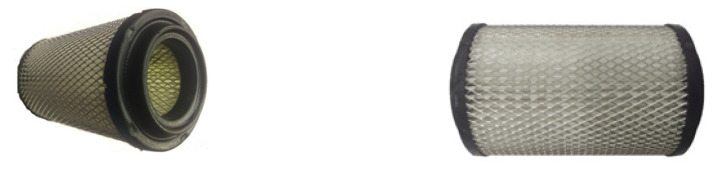
Filter cartridge.

The aim of the research was to determine and compare separation properties—the separation efficiency, filtration performance, pressure drop, and dust absorption coefficient (*k_m_*)—of three different filter materials formed into a cylindrical cartridge (Figure 2), working individually (one-stage system) and in series directly after the cyclone (two-stage system) at the same constant separation speed *υ_F_* = 0.045 m/s.

The subjects of the research were filter inserts (Figure 2) of the same type, with the same dimensions and same separation area (*A_w_* = 0.159 m^2^), but with different filter material chemical composition. Filter material A was cellulose. Filter material B was a composite of cellulose and polyester layer. Filter material C is a composite of cellulose, polyester, and nanofiber layer.

A—cellulose;
B—cellulose and polyester;
C—cellulose, polyester, and nanofibers.

The characteristic parameters of the tested filter materials are summarized in Table 1. Filter materials differed in structure parameter values. The parameters of materials B and C had similar values. Parameters of material A (cellulose) differed significantly from the other two. The permeability of material A was five times, and the thickness was twice that of material B and C. There was a nanofiber layer on the inlet side of filter material C.

The test’s scope of filter materials A, B, and C, working in the “cyclone–filter cartridge” system, without a cyclone, included the determination of the following characteristics, with a fixed value of the *Q_G_* stream:

- separation efficiency *φ_w_ = f*(*k_m_*);
- filtration performance *d_zmax_ = f*(*k_m_*);
- pressure drop Δ*p_w_ = f*(*k_m_*);

where *k_m_*—dust absorption coefficient, defining the total dust mass (*m_z_*), retained and evenly distributed over 1 m^2^ of active filter material surface, which is expressed by the following:

(3) $$k_{m}=\frac{m_{z}}{A_{w}}\;[g/m^{2}].$$

### 2.2. Research Methodology and Conditions

**Figure 3:**
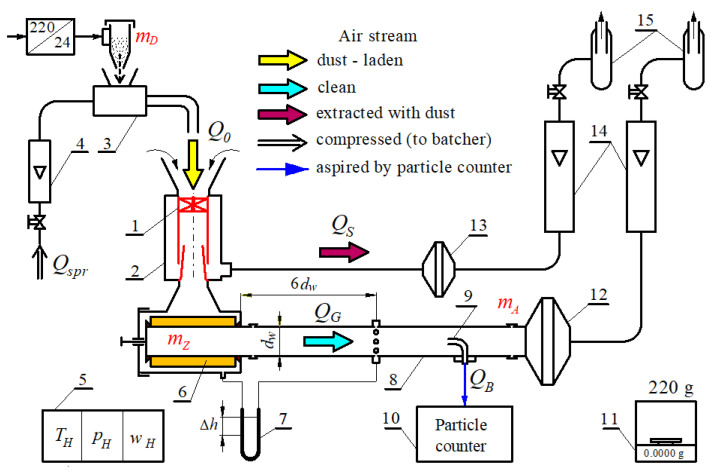
Filter cartridge test stand functional diagram: 1—cyclone; 2—dust collector; 3—dust dispenser; 4—rotameter; 5—instrument for determining air humidity, ambient temperature, and pressure; 6—tested filter cartridge; 7—U-type manometer tube; 8—measuring tube; 9—measuring probe; 10—particle counter; 11—analytical balance; 12 and 13—absolute filter; 14—rotameter; 15—suction fan.

Tests were carried out on the stand (Figure 3), the versatility of which enables the procedures of the basic characteristics of individual cyclones, filter cartridges, and characteristics of cartridges working as the second separation stage after a single cyclone. The cyclone and the filter cartridge located directly behind it form the “filter set”.

**Figure 4:**
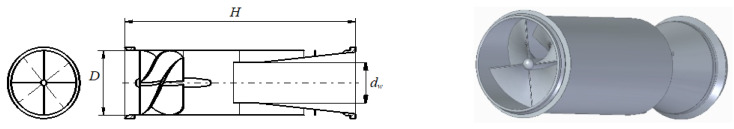
Axial flow cyclone of the “filter set”.

The first tested “cyclone–filter cartridge” filter set filtration stage was a through-flow cyclone made of plastic with the following parameters: internal diameter *D* = 36 mm; total length *H* = 124 mm; outlet pipe internal diameter *d_w_* = 23.5 mm; four-blade steering wheel (Figure 4).

#### 2.2.1. Preliminary Research

**Figure 5:**
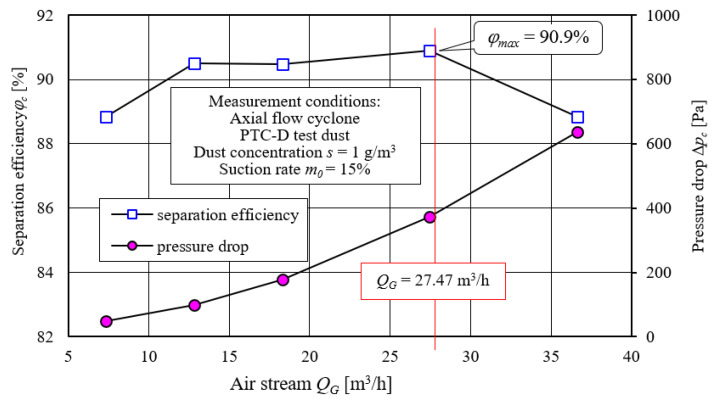
Continuous cyclone separation characteristics.

The purpose of appropriate test condition selection of the “filter set” was determined during the preliminary experimental tests, as follows:

1. Cyclone characteristics in the range of inlet velocity to the cyclone—*υ*_0_ = 2–10 m/s—which corresponds to the exhaust air stream from the cyclone in the range *Q_G_* = 7.32–36.62 m^3^/h (Figure 5).
2. Dust particle size distribution in the air stream before and after the cyclone for the value of the exhaust air stream from the cyclone *Q_G_*, at which the cyclone achieves maximum separation efficiency (Figure 5).

Increase in the air stream *Q_G_* (inlet velocity *υ*_0_) caused a slow increase in separation efficiency. After reaching maximum value of *φ_cmax_* = 90.9% at *Q_G_* = 27.47 m^3^/h, it decreased. Air stream change in the range of *Q_G_* = 7.32–36.62 m^3^/h caused a continuous increase in pressure drop to the value of Δ*p_c_ =* 638 Pa (Figure 5). Such characteristics, *φ_c_* = *f*(*Q_G_*) and Δ*p_c_ = f*(*Q_G_*), were consistent with research results provided in the literature [50,54,56,59].

It was assumed that filter materials tests will be carried out for the main stream, *Q_G_* = 27.47 m^3^/h. This is the value at which the cyclone, during preliminary experimental tests, obtained maximum separation efficiency (Figure 5).

For the assumed air stream value *Q_G_* = 27.47 m^3^/h, the separation speed in the tested cartridge had a value of *υ_Fw_* = 0.045 m/s, so it is within the range of *υ_F_* = 0.03–0.06 m/s, which is expected for truck and special vehicle filters.

**Figure 6:**
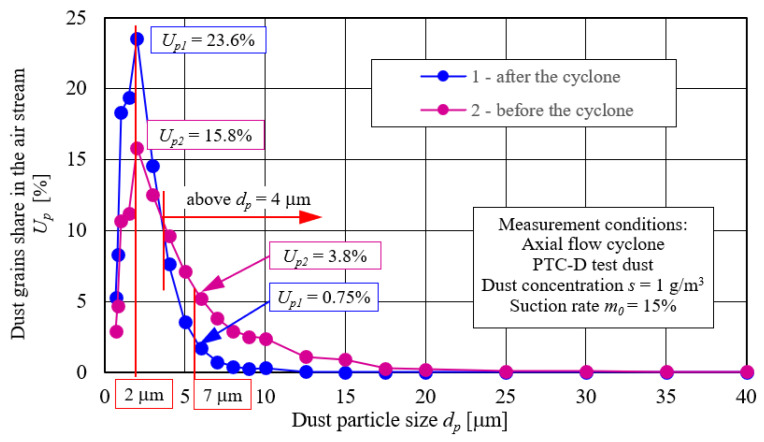
Particle size distribution of PTC-D dust in the air stream before (2) and after the cyclone (1).

Figure 6 shows that dust particle size composition in the air stream flowing from the cyclone shows significant differences in relation to dust supplied to the cyclone. There was an increase in the number of dust grains of smaller sizes (below 4 µm) and a decrease in the number of dust grains with sizes above 4 µm. For example, the share of 2 μm dust grains in the air behind the cyclone increased from *U*_*p*1_ = 15.8% to *U*_*p*2_ = 23.6%, while the share of 7 μm dust grains in the air behind the cyclone decreased from *U*_*p*1_ = 3.8% to *U*_*p*2_ = 0.75%.

**Figure 7:**
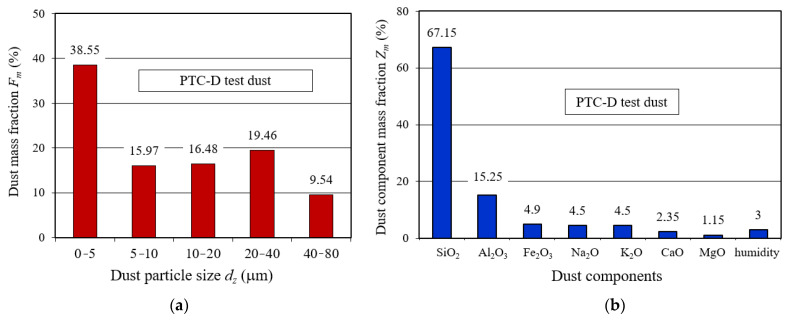
PTC-D test dust: (**a**) mass fraction of individual fractions in the dust; (**b**) components mass fraction in the dust.

In the single-stage system, the PTC-D test dust with particle size composition, shown in line 1 (Figure 6), was dosed directly onto the filter material. In a two-stage system (cyclone–filter cartridge), dust particles that had not been retained by the cyclone was supplied to the filter material, and its particle size distribution is shown in line 2 (Figure 6). The PTC-D test dust, the chemical and fractional composition of which is shown in Figure 7, is used in Poland as a substitute for AC fine test dust.

#### 2.2.2. Basic Research Methodology and Conditions

Tests were carried out with the assumed dust concentration in the air at the inlet of the cyclone *s* = 1 g/m^3^ using the PTC-D test dust, which in Poland is a substitute for the AC fine test dust, whose chemical and fractional composition is shown in Figure 7. During filter material tests without the cyclone, the dust concentration used was *s* = 0.5 g/m^3^.

Test dust was delivered from a vibrating feeder to the dust chamber using a compressed air stream, where it was mixed with inlet air stream *Q*_0_. From the dust chamber, the polluted air was sucked into the cyclone.

Dust separated by the cyclone settled in the dust collector, from which it was removed on an ongoing basis by means of the *Q_S_* suction stream, the value of which was determined from the following dependence, for the assumed extraction rate *m*_0_ = 15%:

*Q_S_* = *Q_G_* × *m*_0_. (4)

Assumed value *m*_0_ = 15% results from the fact that the increase in the suction rate *m*_0_ causes, with a constant air stream (*Q_G_*), an intensive increase in cyclone separation efficiency, but only up to a certain value (*m*_0_ = 12–18%) [66,67]. With a further increase in *m*_0_, the increase in cyclone separation efficiency was not so intense, but there was a significant increase in cyclone flow resistance resulting from the increasing flow velocity.

Dust that hasd not been separated by the cyclone was directed, along with the main air stream (*Q_G_*), to the tested filter cartridge. Directly behind the cartridge there was a measuring line, to which the end of the U-tube water pressure gauge line was connected, used to measure the filter cartridge pressure drop (Δ*p_w_*). The measuring cable ends with a filter that protects the rotameter against dust getting into it, and at the same time is a measuring filter used to collect the *m_AG_* dust mass passed through the test filter cartridge and, consequently, to determine cartridge separation efficiency *φ_w._*

A dust probe tip was mounted centrally in the axis at a distance of 6*d_w_* (where *d_w_* is internal test tube diameter) behind tested filter cartridge. As soon as measurement was started, air and dust were sucked into the Pamas 2132 particle counter. The particle counter sensor registered the number and size of dust grains in the air stream (*Q_G_*) behind the tested filter cartridge. Particle analysis was carried out in the range of zakresie 0.7–100 µm in *i* = 32 measuring intervals, which were limited by diameters (*d_pimin_*–*d_pimax_*).

Before starting filter inserts tests with test dust use, their flow characteristics were performed Δ*p_w_ = f*(*Q_G_*) in the air stream range *Q_G_* = 7.32–36.6 m^3^/h.

Cartridges filtering characteristics—separation efficiency *φ_w_ = f*(*k_m_*) and filtration performance *d_pmax_ = f*(*k_m_*)—were determined indirectly using the gravimetric method. For air stream *Q_G_* = 27.47 m^3^/h, dust mass retained on the tested filter element *m_Fz_*, the absolute filter *m_A_*, and dust mass dosed into the *m_D_* system in successive measurement cycles (*j*) with a specific duration (*τ_p_*) were determined. Measurement duration was determined as *τ_p_* = 120 s in the initial period (I) and *τ_p_* = 240–480 s in the second (II) period of filter cartridges operation. Dust mass retained on tested filter cartridge, the absolute filter, and the dosed mass were determined with an analytical balance with a measuring range of 220 g and an accuracy of 0.1 mg.

During the measurement cycle (60 s before scheduled end of measurement), the procedure for measuring the number and dust grains size in the air after the filter cartridge was started in the particle counter.

At the end of each measurement cycle (*j*), measurements were made in quantities that allowed filter cartridge operating parameters calculation: dust absorption coefficient, filtration efficiency and accuracy, and flow resistance.

1. Filter cartridge pressure drop (Δ*p_fj_*) was determined as the drop in static pressure before and after the filter on the basis of the measured (after the end of dust dosing) height (Δ*h_mj_*) on a U-tube water manometer.
2. Filter element separation efficiency was determined as the quotient of dust mass (*m_Fzj_*) retained by the filter element and dust mass (*m_Fdj_*) supplied to the filter element during the next measurement cycle *j* based on the following:

(5) $$\varphi_{j}=\frac{m_{Fzj}}{m_{Fdj}}=\frac{m_{Fzj}}{m_{Fzj}+m_{Aj}}100\%.$$

3. Dust absorption coefficient (*k_mj_*) of tested filter material was determined from the following:

(6) $$k_{mj}=\frac{\sum_{j=1}^{n}m_{Fzj}}{A_{w}}\;[g/m^{2}].$$

4. Dust grains number (*N_zi_*) in the air stream after the filter (passed through the filter material) was determined in measuring intervals limited by diameters (*d_pimin_–d_pimax_*).
5. Filtration performance was defined as the largest dust grain size (*d_pj_* = *d_pmax_*) in the air stream after the filter.
6. The percentage of individual dust grains fractions in the air after the filter for a given test cycle was calculated from the following:

(7) $$U_{zi}=\frac{N_{zi}}{N_{z}}=\frac{N_{zi}}{\sum_{i=1}^{32}N_{zi}}100\%,$$

where $N_{z}=\sum_{i=1}^{32}N_{zi}$—total dust grains number passed through the filter (from all measurement intervals) in the test cycle.

According to the above methodology, the following separation characteristics were determined: separation efficiency *φ_w_ = f*(*k_m_*), filtration performance *d_zmax_ = f*(*k_m_*), and pressure drop Δ*p_w_ = f*(*k_m_*) of A, B, and C filter elements, operating in a single and two-stage system. Tests were carried out until the filter cartridge reached the assumed permissible resistance value Δ*p_fdop_* = 2.5 kPa.

### 2.3. Filter Materials Test Results Analysis

#### 2.3.1. Research Results Analysis on Filter Materials Flow Characteristics

**Figure 8:**
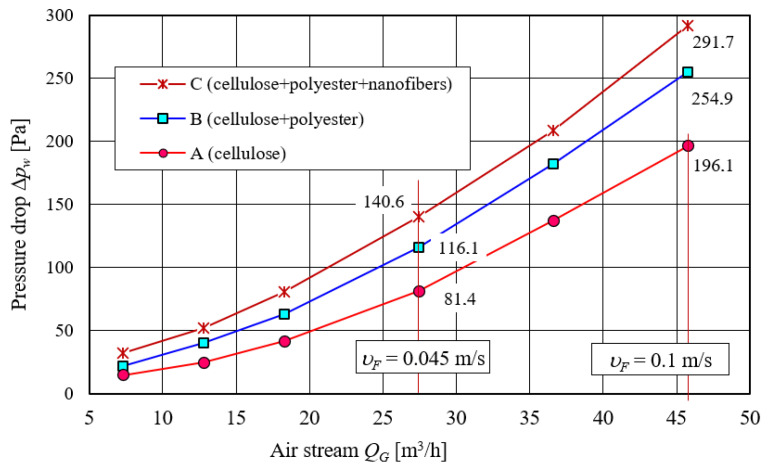
Flow characteristics Δ*p_w_* = *f*(*Q_G_*) of cartridges with filter materials A, B, and C before testing with test dust.

Flow characteristics testing results (Δ*p_w_* = *f*(*Q_w_*)) of cartridges with different types of filter materials are shown in Figure 8. With the increase in air stream, parabolic increase in pressure drop of the filter cartridges occurs, which is consistent with the literature.

The C cartridge with a three-layer (cellulose–polyester–nanofibers) filter material obtained the highest pressure drop value in the entire air stream range. Cellulose filter medium is characterized by the lowest pressure drop, which results from its high permeability in relation to other materials.

#### 2.3.2. Test Results Analysis of Filter Material A (Cellulose)

**Figure 9:**
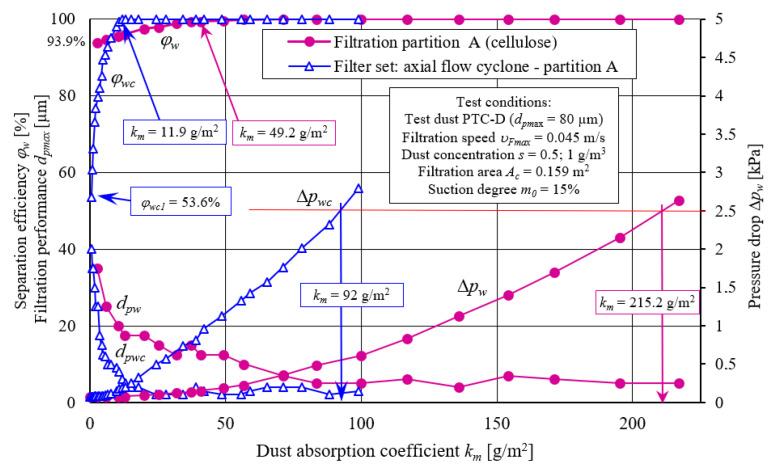
Characteristics: separation efficiency *φ_w_= f*(*k_m_*), pressure drop Δ*p_w_ = f*(*k_m_*), and filtration performance *d_pmax_ = f*(*k_m_*) as a function of dust absorption coefficient (*k_m_*) of filter material A working in the “filter set” as second filtration stage (downstream of the axial flow cyclone) and in a single-stage system.

Figure 9 shows, the dust absorption coefficient (*k_m_*) function characteristics, including filtration efficiency (*φ_w_= f*(*k_m_*)), flow resistance (Δ*p_w_ = f*(*k_m_*)), and filtration accuracy (*d_zmax_ = f*(*k_m_*)) were obtained during filter material A tests (cellulose). Two variants of filter element operation A were tested: in a single-stage system (without cyclone) and in a two-stage system, as the second stage of filtration (after passing through the cyclone).

Obtained characteristics differ significantly in terms of the course and values. Due to the achieved separation efficiency values, tested cartridge working time can be conventionally divided into two periods. The first period (initial filtration) is characterized by small separation efficiency values, which systematically increase (sometimes very rapidly) with the amount of dust mass retained by filtration bed, and thus with an increase in dust absorption coefficient (*k_m_*). Figure 9 shows that first conventional filtration period (initial period) lasts from filtration process start until cartridge filtration material obtains a predetermined filtration efficiency value. In the case of the research conducted, both period separation zones were assumed at the moment of obtaining separation efficiency of *φ* = 99.5% by the inserts. After the first measurement cycle, the effectiveness of A cartridge (cellulose) working without a cyclone reaches value of *φ_w_* = 93.9%, and when working with a cyclone, cartridge efficiency is much lower and amounts to *φ_wc_* = 53.6%—Figure 9. Lower initial separation filtration of cartridge A working after the cyclone is due to the fact that in the air stream flowing from the cyclone (flowing to filter bed of the cartridge) dust grains number of smaller sizes (below 4 mm) is much larger (Figure 6) than in the dust dosed directly on the filter cartridge. Efficiency of retaining dust grains is mainly determined by inertial and direct hooking mechanisms, the importance of which is smaller for small grains.

The initial filtration period of filter element A operating in a two-stage system is characterized by a rapid increase in separation efficiency and a rapid decrease in dust grains size (*d_pmax_*) (from 40 µm to 6 µm) in the *Q_G_* outlet stream and lasts until the *k_m_* = 11.9 g/m^2^ coefficient is reached (Figure 9). Initial filtration period of the A element operating in a single-stage system lasts four times longer, which results from lower separation efficiency intensity increase and ends when filter material reaches dust absorption coefficient *k_m_* = 49.2 g/m^2^. At this time, under conditions of the actual engine operation, a considerable dust mass with large grain sizes of 12.5–35 µm gets along with inlet air. Long duration of the initial period under the actual operation conditions of the filter has an adverse effect on the accelerated engine components wear.

Small-sized dust particles quickly form dendrites on the fibers, which fill free spaces (pores) in the fibrous bed. For this reason, filter element working after cyclone reaches determined separation efficiency value faster than *φ* = 99.5%. From that moment, conventional period of basic filtration begins, which is characterized by separation efficiency stabilization at the level of *φ* = 99.5–99.98% (Figure 9).

After first measurement cycle, there are dust grains with the maximum dimensions of *d_pmax_* = 40 µm in the air behind the A filter cartridge, tested in the cyclone unit (Figure 9). For the test element without a cyclone, the grain size *d_pmax_* = 35 µm. However, along with dust absorption coefficient increase (*k_m_*), the grain diameters of *d_zmax_* assume smaller and smaller values. They faster decrease in diameter occurs for contribution tested in set with a cyclone (Figure 9). After reaching coefficient *k_m_* = 11.9 g/m^2^, grain size stabilizes at the level of *d_pmax_* = 2–4 µm (Figure 9). For the A paper insert working without a cyclone, dust grain size stabilization occurs after a longer period, after reaching *k_m_* = 49.2 g/m^2^, and at a higher level of *d_pmax_* = 5–12.5 µm. In real operation conditions, this undoubtedly has a large impact on increased engine components wear.

**Figure 10:**
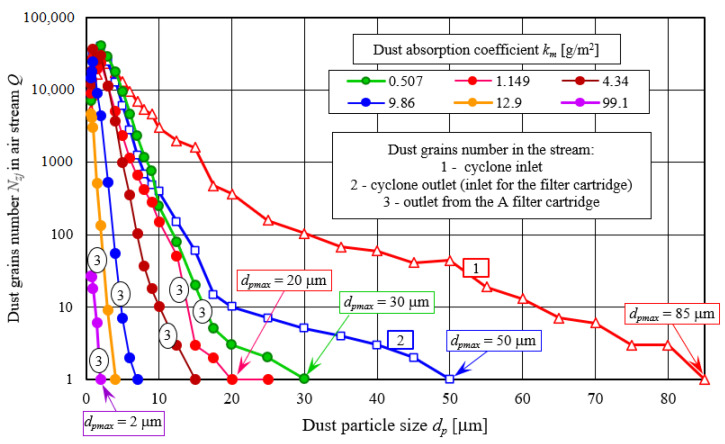
Dust grains number in inlet stream to the cyclone (1), outlet stream from the cyclone (2), and outlet stream from the filter element (3) in individual size ranges for different values of dust absorption coefficient (*k_m_*) during filter element A tests (cellulose) in set operation “cyclone–cartridge”.

**Figure 11:**
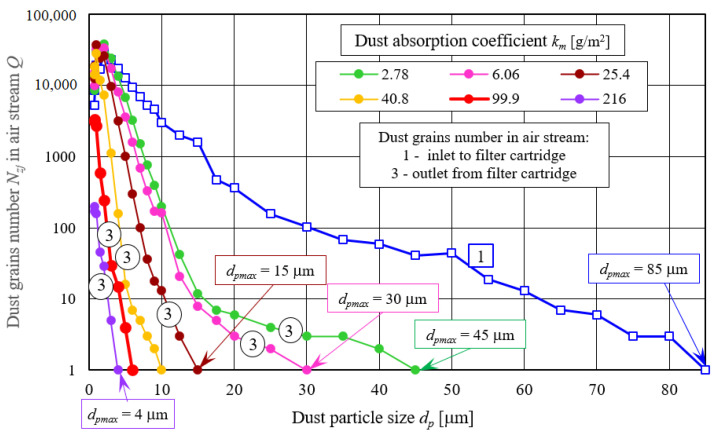
Dust grains number in inlet stream to cyclone (1) and outlet stream from filter element (3) in individual size ranges for different dust absorption coefficient (*k_m_*) values during filter element A tests (cellulose) working without cyclone.

At the same time, along with separation efficiency increase, the decrease in total number *N_zp_* of dust grains in the air behind filter element was recorded in each subsequent measurement cycle—Figure 10. Number of dust particles *N_zj_* for subsequent measurement cycles (for different dust absorption coefficient values *k_m_*) in the outlet stream from filter cartridge A (cellulose) working in the “cyclone–cartridge” set is shown in Figure 10, and for cartridge without cyclone is shown in Figure 11. There is a clear relationship between the grain size *d_p_* and their *N_z_* number. As dust grains size increases, their number decreases until they disappear completely. Grains (or a single grain) in the last dimensional range have the largest size *d_p_* = *d_pmax_* (Figure 10). It was assumed that dust grain with the largest size (*d_pmax_*) in exhaust air stream from test filter cartridge expresses filtration performance of filter material in the next measuring cycle (*j*). Additionally, for comparison, a number of test dust grains corresponding to the individual size ranges are shown, in a cyclone inlet stream (line 1) and in a cyclone outlet stream–inlet to filter cartridge (line 2—Figure 10).

Dust grains number *N_z_* in the exhaust air stream *Q_G_* from filtering cartridge A, which decreases with each measurement, causes a change in dust particle size distribution in individual size ranges. Figure 11 shows dust grains particle size composition in the *Q_G_* stream downstream of filter element for subsequent measurements (different *k_m_* coefficient values), which were compared with dust particle size composition in cyclone inlet stream, which is test dust.

**Figure 12:**
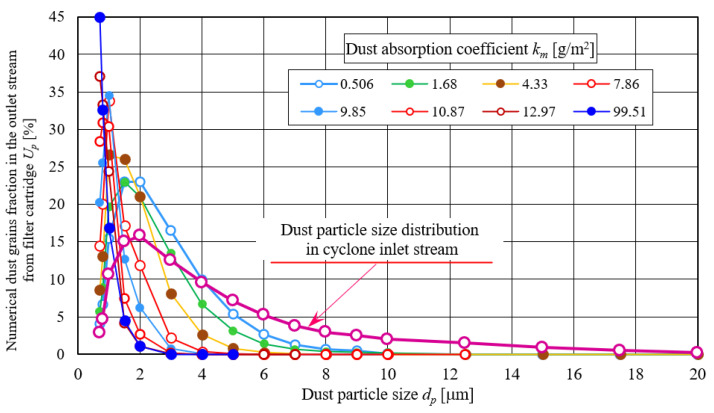
Dust grains particle size distribution in *Q_G_* stream downstream of the filter element in individual size ranges for different *k_m_* coefficient values.

Numerical fractions of *U_p_* dust grains in the outlet stream from filter cartridge take larger and larger values for increasingly larger grain sizes, followed by their decrease. This changes nature in numerical shares of *U_p_* is similar to particle size composition of dust dosed into the cyclone—test dust (Figure 12). Maximum test dust grains number is *U_pmax_* = 15.8% for dust grains with size *d_p_* = 2 µm. For the first measurement (*k_m_* = 0.506 g/m^2^), maximum dust grains share in the air behind filter element takes greater value, *U_pmax_* = 23.1%. In subsequent measurements, maximum particle size assumes ever greater values, but for ever smaller dust particles. For *k_m_* = 9.85 g/m^2^ (measurement no. 25), numerical dust grains share has value *U_pmax_* = 34.5% for *d_p_* = 1 µm. In last measurement (*k_m_* = 99.5 g/m^2^), maximum dust grains number *U_pmax_* = 44.9% occurs for *d_p_* = 0.7 µm, while dust grains share above *d_p_* = 2 µm is only *U_p_* = 1.1%.

The above phenomenon is caused by the fact that tested filter bed (A—cellulose) is characterized by high permeability. In initial filtration period, mainly larger dust grains are retained. However, as dust accumulates in filter bed, voids (pores) fill up in it.

Along with the increase in dust mass retained by the filter material (increase in dust absorption coefficient *k_m_*), pressure drop Δ*p* of tested filter inserts is systematically growing all the time. This is due to the dendrites formed on the fibers and growing to large sizes, which fill free spaces between the fibers.

The expanding dendrites formed on the fibers resembled trees. The farthest particles are exposed, to a large extent, to air flow, thus causing greater pressure drops. Dust particles, which are stopped by inertial mechanism, are deposited on fiber front surface and form tightly packed sediments. In this case, fewer particles are exposed to flowing stream and resulting pressure drop is lower. In addition, small-sized particles adhere more closely to each other and are more flow-blocking than larger-sized particles because they have larger area to volume ratio *a_g_*. Consequently, they achieve greater pressure drop per unit volume/molecules mass.

As a result of this, aerosol flows through narrow slots with increased velocity, which is direct cause of pressure drop increase. Expanding dendrites cause retention of smaller and smaller dust grains, which should be explained by continuous increase in separation efficiency and performance.

Increase in cartridge pressure drop, operating in a two-stage system (cyclone-filter cartridge), is more intense, despite smaller dust mass retained by the bed. This is because small-sized dust particles form dendrites on the fibers that grow faster when bed is loaded with smaller particles. Small-sized dust grains, the number of which is much greater when cartridge is operated with a cyclone, penetrate into filter paper structure much more easily and fill it more tightly compared with grains with larger diameters. Free spaces created between the anchored dust particles of small sizes are much smaller than in case of large dust particles, which makes aerosol flow velocity through them higher, and thus pressure drop increases.

**Figure 13:**
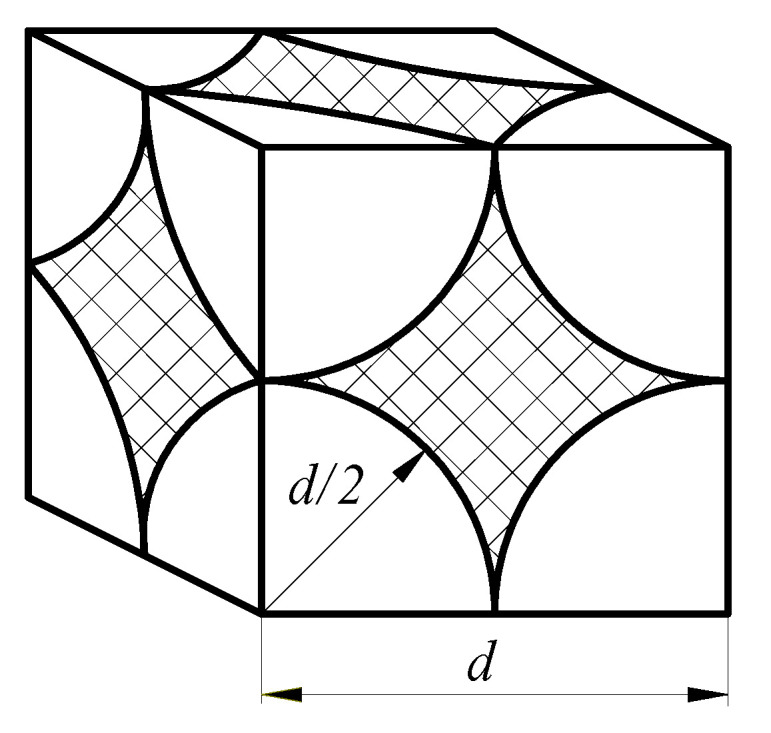
Same diameter spherical particles bed model.

Mineral dust grains have irregular, lumped shapes. Therefore, for simplicity, during theoretical considerations, equivalent grain diameters are assumed. Most often, it is grain diameter, which is shaped like a sphere. Figure 13 shows a section of deposit model in cube form with a side (*d*) made of regularly arranged spheres, where *d* is also sphere diameter.

For the model shown in Figure 12, space volume (*V*_1_) between spherical particles equals cube volume (*V*) minus volume *V_2_*, occupied by particles according to the following equations:

*V*_1_ = *V* − *V*_2_ (8)

(9) $$V_{1}=d^{3}-(\frac{4}{3}\pi (\frac{d}{2}{)}^{3})$$

(10) $$V_{1}=d^{3}\;(1-\frac{\pi }{6}).$$

Presented Equation (10) shows that space volume *V*_1_ between spherical dust grains depends only on grain diameter (*d*) in the third power. After combining successive volumes *V*_1_ between the particles, a channel is obtained, with an assumed diameter (*d_ε_*), through which air flows. According to Equation (10), for particles with smaller diameters (*d*), space volume between particles (*V*_1_) assumes smaller and smaller values. For a bed composed of particles with equivalent diameter of *d* = 80 µm, space volume (*V*_1_) takes value, as follows:

$$V_{1(80)}=80^{3}\;(1-\frac{\pi }{6})$$

*V*_1(80)_ = 244,053 µm^3^.

Accordingly, space volume (*V*_1_) between particles with equivalent diameter *d* = 40 µm (50% smaller) has value *V*_1(40)_ = 30,506 µm^3^, and thus is 8 times smaller. For the diameter of *d* = 8 µm, space volume *V*_1(8)_ is 1000 times smaller, and for *d* = 2 µm, *V*_1(2)_ is 64,000 times smaller.

For the same air stream value *Q* (separation velocity *υ_F_*), flowing through the filter bed made of particles with smaller diameters, the actual velocity (*υ_ε_*) in the channels between the particles assumes higher and higher values. Therefore, pressure drop (Δ*p*) of the filter bed loaded with small-diameter dust particles will assume even greater values in accordance with modified Darcy Weisbach formula.

(11) $$\Delta p=\lambda \left(Re\right)\frac{g_{w}}{d_{\varepsilon }}\frac{\rho }{2}\upsilon_{\varepsilon }^{2},$$

where *ρ*—fluid density; *λ*—friction coefficient; *g_w_*—filter bed thickness; *d_ε_*—conventional channels diameter formed by particles; *υ_ε_*—actual velocity in channels between particles.

Moreover, small-sized dust grains have a higher surface to volume ratio. Therefore, they get a greater pressure drop per unit volume–particles mass. Spherical particle ratio surface (*A*_c_) to its volume (*V_c_*) expresses the following relationship:

(12) $$a_{g}=\frac{A_{c}}{V_{c}}=\frac{6}{d}$$

For example, for particles with an equivalent diameter: *d_z_* = 0.7, 1.0, 2.0, 10, 20, 50, 80 µm, the particle surface area to volume ratios are: *a_g_* = 8.57, 6, 3, 0.6, 0.3, 0.12, 0.075, respectively.

Conducted experimental studies results (Figure 9) showing a faster increase in pressure drop of fibrous filters for small particles confirm model test results obtained by the authors [62,64,65,68,69,70]. During model tests of the filtration process in the bed, two test dusts were used—monodisperse and polydisperse dust—with grain diameters from 1 µm to 10 µm.

Test results presented in Figure 9 clearly prove that filter cartridge (A—cellulose) working in series behind the cyclone is characterized by over three times lower dust absorption than the same cartridge traditionally operating in a single-stage system. This has a decisive impact on reaching speed limit resistance, and thus on vehicle’s mileage. Reaching permissible resistance by the filter is a condition for its maintenance—filter cartridge replacement. Although the filter element working in cyclone unit is characterized by lower absorption capacity, the working time of “multicyclone–element” filtration unit until a permissible resistance is reached is much longer than that of same single element working, which is main advantage of two-stage filtration.

**Figure 14:**
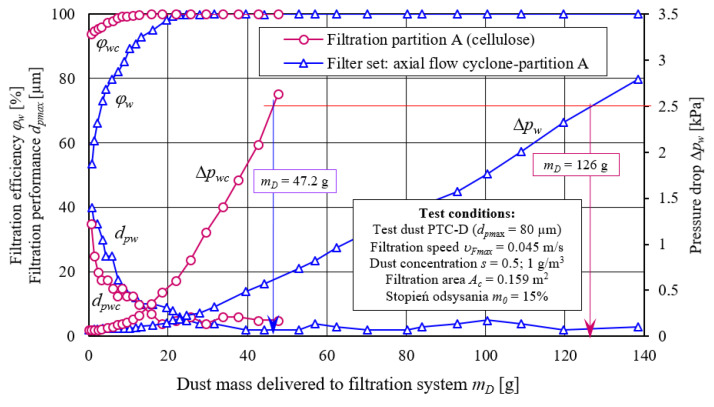
Characteristics: separation efficiency *φ_w_= f*(*m_D_*), pressure drop Δ*p_w_ = f*(*m_D_*), filtration performance *d_pmax_ = f*(*m_D_*) as dust mass function *m_D_* supplied to filter element operating in a single-stage and two-stage system “cyclone–cartridge”.

Figure 14 shows the following characteristics: separation efficiency *φ_w_= f*(*m_D_*), pressure drop Δ*p_w_ = f*(*m_D_*), and filtration performance *d_pmax_ = f*(*m_D_*), as dust mass function *m_D_* is supplied to the filter element A, operating in one-stage and two-stage “cyclone–cartridge” separation system.

Presented characteristics show that working time of “cyclone–cartridge” unit reaching permissible resistance (2.5 kPa) is three times longer than that of the same single working cartridge. It happens despite the fact that filter element in two-stage system obtains much lower dust absorption.

When an inertial filter (multi-cyclone) is used as the first filtration stage before the paper filter cartridge, the vast majority of supplied dust (in case of tested set—about 80% of dust mass) is retained by the multi-cyclone. Thus, only 20% of dust mass, which was introduced into the “multicyclone–partition” separation system, goes to filter cartridge. Cyclones are characterized by the fact that they are able to trap large dust masses from the stream of flowing aerosol, but with low efficiency and accuracy. Retained dust is accumulated in settling tank, which must be periodically removed, or it is continuously removed outside the vehicle by dust extraction system. Therefore, multicyclone pressure drop (at a constant stream of flowing air) is unchanged, in contrast to barrier filter materials, where dust mass retention causes an increase in pressure drop.

#### 2.3.3. Test Results Analysis of Filter Material C (Cellulose, Polyester, Nanofiber Layer)

**Figure 15:**
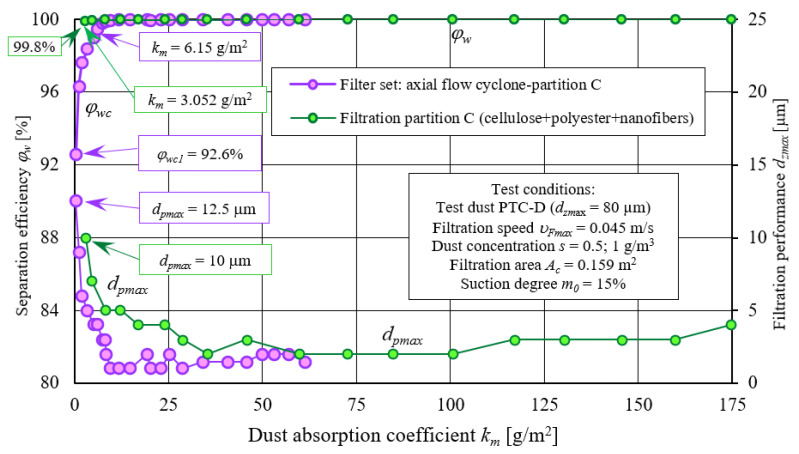
Characteristics: separation efficiency *φ_w_= f*(*k_m_*) and filtration performance *d_pmax_ = f*(*k_m_*) as a function of the dust absorption coefficient *k_m_* of filter material C working in the “filter set” as the second filtration stage (after through cyclone) and in single-stage system—no cyclone.

**Figure 16:**
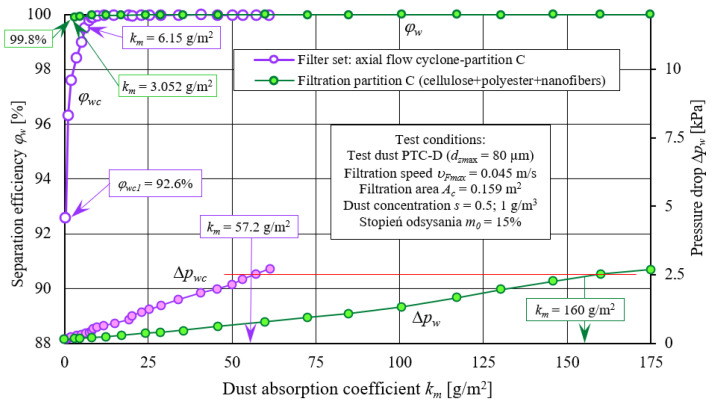
Characteristics: separation efficiency *φ_w_= f*(*k_m_*) and pressure drop Δ*p_w_ = f*(*k_m_*) as a function of dust absorption coefficient *k_m_* of filter material C working in the “filter set” as the second filtration stage (after through cyclone) and in a single-stage system—cyclone barrels.

Figure 15 and Figure 16 show characteristics of the following: separation efficiency (*φ_w_= f*(*k_m_*)), filtration performance (*d_pmax_ = f*(*k_m_*)), and pressure drop (Δ*p_w_ = f*(*k_m_*)) as a function of dust absorption coefficient *k_m_* of filter material C tested in the “set filtration“ as a second filtration stage downstream of the through-flow cyclone and without the cyclone. Tests were performed as a function of dust absorption coefficient (*k_m_*) according to same methodology as filter element A tests. Filter material C is a composite of cellulose, polyester, and nanofibers layer (Table 1).

Obtained characteristics show that C filter cartridge operating in “filter set”, as the second filtration stage after through-cyclone, obtains assumed pressure drop of 2.5 kPa much faster (three times) than when it works in a single-stage system (without a cyclone). In case of material C, on filter bed surface there is nanofibers layer on which surface filtration and faster accumulation of dust layer take place. Permeability of dust layer made of small dust grains is much lower due to their tight mutual adhesion and easier penetration into the structure of the filter paper compared with grains with larger diameters. For this reason, pressure drop increases more intensively, and a set value of 2.5 kPa for the filter cartridge C, working in a set with a cyclone, reaches a dust absorption coefficient of *k_m_* = 57.2 g/m^2^, and without a cyclone, at *k_m_* = 160 g/m^2^. Thus, it confirms results obtained for the filter element A.

Maximum dust grains diameters (*d_pmax_*) in the air stream downstream of C cartridge working in “filter set” initially assume considerable dimensions (measurement no. 1 *d_pmax_* = 12.5 µm) which results from greater proportion of small-diameter dust grains in the air, but with the accumulation of dust in the bed, they quickly assume lower and lower values. After reaching absorption coefficient *k_m_* = 6.15 g/m^2^, they are in range *d_pmax_* = 1–3 µm. In the same period, in air stream downstream of the C element working in a single-stage system (without a cyclone), maximum dust grains diameters *d_zmax_* assume much greater values in the range *d_pmax_* = 2–10 µm.

#### 2.3.4. Test Results Comparative Analysis for A, B, and C Filter Materials for Two-Stage Filtration

**Figure 17:**
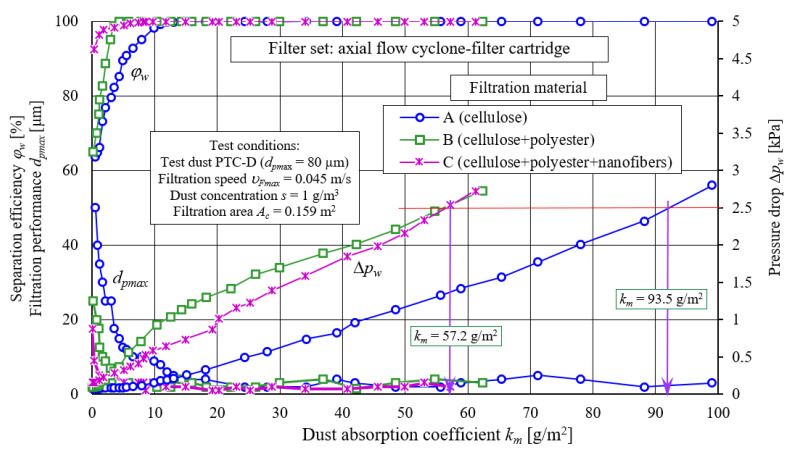
Characteristics: separation efficiency *φ_w_= f*(*k_m_*), filtration performance *d_pmax_ = f*(*k_m_*) and pressure drop Δ*p_w_ = f*(*k_m_*) as a function of dust absorption coefficient *k_m_* of filter materials A, B and C tested in “filtration set” as the second filtration stage after axial flow cyclone.

Figure 17 presents a comparative characteristics analysis: separation efficiency *φ_w_= f*(*k_m_*), filtration performance *d_pmax_ = f*(*k_m_*), and pressure drop Δ*p_w_ = f*(*k_m_*), as a function of dust absorption coefficient *k_m_* of filter materials A, B, and C tested in the “filter assembly” as the second stage of separation downstream of through-cyclone. Characteristics were performed as a function of dust absorption coefficient (*k_m_*) according to the same methodology, maintaining same test conditions.

Presented tested filter materials characteristics A, B, and C (Figure 17) differ in terms of values, but are similar in terms of their course. These differences result from different chemical composition of filter material from which the cartridges are made (Table 1). Filter materials B and C, which are cellulose, polyester, and nanofiber composites, achieve much earlier assumed separation efficiency of *φ_w_* = 99.5% than filter cartridge made of material A (cellulose), and thus the initial filtration period of the B and C filter material lasts a much shorter time. Maximum dust grains diameters in the air stream after the B and C inserts assume much smaller sizes (*d_pmax_* = 25 µm and *d_pmax_* = 17.5 µm, respectively) than after the A insert—*d_pmax_* = 50 µm. This makes use of B and C filter materials more favorable in real engine operating conditions.

As dust mass retained on filter cartridges increases, value and pressure drop intensity of filter cartridges increase, resulting from different properties of filter materials A, B, and C (Table 1). The least intense (almost linear) increase in pressure drop was recorded for the insert A, which obtained determined pressure drop value Δ*p_w_* = 2.5 kPa for *k_m_* = 99.5 g/m^2^.

The most intense (parabolic)—but only in the first filtration period—increase pressure drop was recorded for the B element. With particle deposition increase on sample B (cellulose and polyester), there is a slow exponential increase in pressure drop, until a linear trend is reached, the slope of which is the same as pressure drop of linear A trend (cellulose) and C (cellulose–polyester–nanofibers) cartridge—Figure 16. Determined pressure drop value Δ*p_w_* = 2.5 kPa, the B and C cartridges are achieved with absorption coefficient *k_m_* = 57.2 g/m^2^, which is 50% less than cartridge A. This is due to the fact that cartridges B and C use composite filter materials, unlike cartridge A, which has a conventional cellulose bed.

After the first measurement, maximum dust grains diameters in air stream after the B and C inserts assume much smaller sizes (*d_pmax_* = 25 µm and *d_pmax_* = 17.5 µm, respectively) than after the A insert—*d_pmax_* = 50 µm. This makes the use of B and C filter materials more favorable for real engine operating conditions. However, it is related to increased frequency of filter inserts replacement, resulting from a more intensive increase in pressure drop and permissible resistance achievement Δ*p_fdop_*.

## 3. Conclusions

This paper presents an experimental filtration properties evaluation—of the separation efficiency, filtration performance, pressure drop, and dust absorption coefficient (*k_m_*)—of three different filter materials, formed into a cylindrical cartridge, which worked individually (one-stage system) and directly after the axial flow cyclone (two-stage system) with the same constant separation speed *υ_F_* = 0.045 m/s. The aim of the study was to compare the characteristics of several filter media types when loaded with different dust grain size range and to show that, if dust with small grain sizes flows onto the filter material, it has an impact on separation process and filter material characteristics. Such a case occurs when filter element works in a two-stage system of air inlet filtration to the “multi-cyclone–porous partition” engine. During the experiment, original methodology was used, which consisted in the fact that filter cartridge material, which worked behind the cyclone, was filled with dust. It was stripped of large grains, whose grain size composition was changed and shaped as a result of the actual air filtration process in the cyclone.

Analysis was performed in the context of using research results to design paper filters partitions for trucks. Air filter selection for a truck engine requires dust absorption coefficient (*k_m_*) knowledge of the filter material working directly after the inertial filter as the second filtration stage.

As a result of the research, the following conclusions were formulated:

1. Filter materials working in the “cyclone–filter cartridge” system achieve an absorption coefficient 2–4 times lower than the same materials working in a single-stage filtration system. It is undoubtedly influenced by dust particle size composition (large share of small dust grains). This is related to a more intensive pressure drop increase and has a direct impact on filter operation time limited by permissible resistance achievement, and thus on vehicle mileage.
2. Low (*φ* = 63–92%) tested filter materials A, B, and C efficiency and large (*d_zmax_* = 17.5–50 μm) dust grains presence in purified air in the initial operation period is fibrous materials characteristic feature which has been demonstrated. In real operating conditions of air filter and engine, this may have a direct impact on accelerated engine couplings wear that cooperate in the form of a tray, especially piston–piston rings–cylinder (P–P–C).
3. Initial filter materials operation period is significantly shortened when they are used in the “cyclone–filter cartridge” system. Filtration materials, which are cellulose, polyester, and nanofiber composites, are characterized by an initial filtration time several times shorter compared with the standard cellulose material.
4. Developed research methodology allows for experimental determination of basic filter materials characteristics for second air filtration stage with any structure parameters and in a wide changes range in filtration conditions corresponding to air filter operation in dusty conditions.
5. Research methodology originality lies in the fact that filter material is contaminated with dust, the chemical and grain composition of which has been changed and shaped as a result of the actual air filtration process in the cyclone.
6. It has been found that installing a pre-filter (multi-cyclone) before the main filter can improve entire filtration system performance by extending the main filter’s service life, despite a decrease in its absorption capacity due to small-sized dust grains inflow.

## Figures and Tables

**Table 1:** Tested filtration materials parameters, according to the manufacturer’s data.

Parameters	Filter Paper Identification		
	A	B	C
Filtration material	Cellulose	90% cellulose and polyester	Cellulose, polyester, and nanofibers
Permeability *q_p_* [m^3^/m^2^/h] at 200 Pa	3015	685	540
Permeability *q_p_* [dm^3^/m^2^/s]	838	190	150
Grammage *g_m_* [g/m^2^]	121	135	130
Thickness *g_z_* [µm]	610	360	300

## Data Availability

Data sharing is not applicable to this article.

## References

[1] Bojdo N., Filippone A. Effect of desert particulate composition on helicopter engine degradation rate. Proceedings of the 40th European Rotorcraft Forum.

[2] Smialek J.L., Archer F.A., Garlick R.G. (1994). Turbine airfoil degradation in the persian gulf war. JOM.

[3] Haig C., Hursthouse A., McIlwain S., Sykes D. (2014). The effect of particle agglomeration and attrition on the separation efficiency of a Stairmand cyclone. Powder Technol..

[4] Barris M.A. (1995). Total Filtration™: The Influence of Filter Selection on Engine Wear, Emissions, and Performance.

[5] Jaroszczyk T., Fallon S.L., Liu Z.G., Heckel S.P. (1999). Development of a Method to Measure Engine Air Cleaner Fractional Efficiency. SAE Trans..

[6] Long J., Tang M., Sun Z., Liang Y., Hu J. (2018). Dust Loading Performance of a Novel Submicro-Fiber Composite Filter Medium for Engine. Materials.

[7] Treuhaft M.B. (1993). The Use of Radioactive Tracer Technology to Measure Engine Ring Wear in Response to Dust Ingestion. SAE Trans..

[8] Wróblewski P. (2020). Technology for Obtaining Asymmetries of Stereometric Shapes of the Sealing Rings Sliding Surfaces for Selected Anti-Wear Coatings. Event: SAE Powertrains, Fuels & Lubricants Meeting.

[9] Wróblewski P., Iskra A. (2016). Geometry of shape of profiles of the sliding surface of ring seals in the aspect of friction losses and oil film parameters. Combust. Engines..

[10] Bojdo N., Filippone A. (2019). A Simple Model to Assess the Role of Dust Composition and Size on Deposition in Rotorcraft Engines. Aerospace.

[11] Schaeffer J.W., Olson L.M. (1998). Air Filtration Media for Transportation Applications. Filtr. Sep..

[12] Jaroszczyk T., Pardue B.A., Heckel S.P., Kallsen K.J. Engine air cleaner filtration performance—Theoretical and experimental background of testing. Proceedings of the AFS Fourteenth Annual Technical Conference and Exposition.

[13] Pinnick R.G., Fernández G., Hinds B.D., Bruce C.W., Schaefer R.W., Pendleton J.D. (1985). Dust Generated by Vehicular Traffic on Unpaved Roadways: Sizes and Infrared Extinction Characteristics. Aerosol Sci. Technol..

[14] Barbolini M., Di Pauli F., Traina M. (2014). Simulation der Luftfiltration zur Auslegung von Filterelementen. MTZ Mot. Z..

[15] Dziubak T. (1990). Zapylenie powietrza wokół pojazdu terenowego. Wojsk. Przegląd Tech..

[16] Burda S., Chodnikiewicz Z. (1962). Konstrukcja i badania pyłowe filtrów powietrza silnika czołgowego. Bull. Mil. Univ. Technol..

[17] Bojdo N., Rotorcraft Engine Air Particle Separation (2012). A Thesis Submitted to the University of Manchester for the Degree of Doctor of Philosophy in the Faculty of Engineering and Physical Sciences. https://www.escholar.manchester.ac.uk/uk-ac-man-scw:183545.

[18] Szczepankowski A., Szymczak J., Przysowa R. The Effect of a Dusty Environment Upon Performance and Operating Pa-rameters of Aircraft Gas Turbine Engines. Proceedings of the Specialists’ Meeting—Impact of Volcanic Ash Clouds on Military Operations (NATO AVT-272-RSM-047).

[19] Zhang T., Choi S., Ahn S., Nam C., Lee G. (2021). Enclosure Design for Brake Wear Particle Measurement Using Computational Fluid Dynamics. Energies.

[20] Timmers V.R.J.H., Achten P.A.J. (2016). Non-exhaust PM Emissions from Electric Vehicles. Atmos. Environ..

[21] Lee P.G., Jung S.P., Park M.K., Sim S.K. (2018). Study on Dust Characteristics of Brake Pads Using Brake Dynamometer. Korean Soc. Automot. Eng. Spring Conf..

[22] Luo H., Zhou W., Jiskani I.M., Wang Z. (2021). Analyzing Characteristics of Particulate Matter Pollution in Open-Pit Coal Mines: Implications for Green Mining. Energies.

[23] Wanjun T., Qingxiang C. (2018). Dust distribution in open-pit mines based on monitoring data and fluent simulation. Environ. Monit. Assess..

[24] Li L., Zhang R., Sun J., He Q., Kong L., Liu X. (2021). Monitoring and prediction of dust concentration in an open-pit mine using a deep-learning algorithm. J. Environ. Health Sci. Eng..

[25] Rieger M., Hettkamp P., Löhl T., Madeira P.M.P. (2019). Effcient Engine Air Filter for Tight Installation Spaces. ATZ Heavy Duty Worldw..

[26] Tian X., Ou Q., Liu J., Liang Y., Pui D.Y. (2019). Influence of pre-stage filter selection and face velocity on the loading characteristics of a two-stage filtration system. Sep. Purif. Technol..

[27] Sanders R., Bühler A., Durst M., Moser N., Pelz A. (2007). Effiziente Motorluftfiltration durch den Einsatz von Nanofasern. MTZ Mot. Z..

[28] Schulze M., Taufkirch G. (1991). Papierluftfilter Nutzfahrzeugen. MTZ Mot. Z..

[29] Sun Z., Liang Y., He W., Jiang F., Song Q., Tang M., Wang J. (2019). Filtration performance and loading capacity of nano-structured composite filter media for applications with high soot concentrations. Sep. Purif. Technol..

[30] Liu B., Zhang S., Wang X., Yu J., Ding B. (2015). Efficient and reusable polyamide-56 nanofiber/nets membrane with bimodal structures for air filtration. J. Colloid Interface Sci..

[31] Jaroszczyk T., Wake J., Fallon S.L., Connor M.J. (1996). Development of Motor Vehicle Ventilation System Particulate Air Filters.

[32] Durst M., Klein G., Moser N. (2005). Filtration in Fahrzeugen, Mann+Hummel.

[33] Maddineni A.K., Das D., Damodaran R.M. (2019). Numerical investigation of pressure and flow characteristics of pleated air filter system for automotive engine intake application. Sep. Purif. Technol..

[34] Dziubak T., Dziubak S.D. (2020). Experimental Study of Filtration Materials Used in the Car Air Intake. Materials.

[35] Long J., Tang M., Liang Y., Hu J. (2018). Preparation of fibrillated cellulose nanofiber from lyocell fiber and its application in air filtration. Materials.

[36] Sakthivel S., Ezhil A.J., Ramachandran T. (2014). Development of Needle-Punched Nonwoven Fabrics from Reclaimed Fibers for Air Filtration Applications. J. Eng. Fibers Fabr..

[37] Wang Q., Lin X., Chen D.-R. (2016). Effect of dust loading rate on the loading characteristics of high efficiency filter media. Powder Technol..

[38] Bugli N.J., Green G.S. (2005). Performance and Benefits of Zero Maintenance Air Induction Systems. Proceedings of the SAE World Congress, Detroit, MI, USA, 11–14 April 2005.

[39] Trautmann P., Pelz A., Durst M., Moser N. High Performance Nanofibre Coated Filter Media for Engine Intake. Proceedings of the Air AFS 2005 Conference and Expo.

[40] Heikkilä P., Sipilä A., Peltola M., Harlin A., Taipale A. (2007). Electrospun PA-66 Coating on Textile Surfaces. Text. Res. J..

[41] Jaroszczyk T., Fallon S.L., Schwartz S.W. (2008). Development of High Dust Capacity, High Efficiency Engine Air Filter with Nanofibers. J. KONES Powertrain Transp..

[42] Grafe T., Gogins M., Barris M., Schaefer J., Canepa R. Nano fibers in filtration applications in transportation. Proceedings of the Filtration 2001 International Conference and Exposition of the INDA.

[43] Tian X., Ou Q., Liu J., Liang Y., Pui D.Y. (2019). Particle loading characteristics of a two-stage filtration system. Sep. Purif. Technol..

[44] Pei C., Ou Q., Yu T., Pui D.Y. (2019). Loading characteristics of nanofiber coated air intake filter media by potassium chloride, ammonium sulfate, and ammonium nitrate fine particles and the comparison with conventional cellulose filter media. Sep. Purif. Technol..

[45] Gervais P.-C., Poussier S., Bardin-Monnier N., Karcher G., Thomas D. (2014). Combination of Single-Photon Emission and X-Ray Computed Tomography to visualize aerosol deposition in pleated filter. Sep. Purif. Technol..

[46] Erdmannsdörfer H. (1971). Leistungsmöglichkeiten von Papierfiltern zur Reinigung der Ansaugluft von Diselmotoren. MTZ Mot. Z..

[47] Taufkirch G., Mayr G. (1984). Papierluftfilter für Motoren in Nutzfahrzeugen. MTZ Mot. Z..

[48] Bugli N.J. (2001). Automotive Engine Air Cleaners—Performance Trends.

[49] Mrad W., Theron F., Joubert A., Zgheib N., Le Coq L. (2021). Local variations of air velocity in the vicinity of filter pleats in transitional airflow regime—Experimental and numerical approaches. Sep. Purif. Technol..

[50] Chu K., Chen J., Yu A. (2016). Applicability of a coarse-grained CFD–DEM model on dense medium cyclone. Miner. Eng..

[51] Zhang W., Zhang L., Yang J., Hao X., Guan G., Gao Z. (2019). An experimental modeling of cyclone separator efficiency with PCA-PSO-SVR algorithm. Powder Technol..

[52] Baltrėnas P., Chlebnikovas A. (2015). Experimental research on the dynamics of airflow parameters in a six-channel cyclone-separator. Powder Technol..

[53] Jaroszczyk T., Ptak T. Experimental Study of Aerosol Separation Using a Minicyclone. Proceedings of the 10th Annual Powder and Bulk Solids Conference.

[54] Dziubak T. (2018). Potential Design Improvements of A Reverse Flow Mini-Cyclone with a Tangential Inlet. Chem. Process. Eng..

[55] Dziubak T. (2017). Problems of dust removal from multi-cyclones of engine air cleaners in cross-country motor vehicles. Arch. Automot. Eng..

[56] Dziubak T. (2020). Experimental research on separation efficiency of aerosol particles in vortex tube separators with electric field. Bull. Pol. Acad. Sci. Tech. Sci..

[57] Jaroszczyk T., Fallon S.L., Pardue B.A. (2002). Analysis of Engine Air Cleaner Efficiency For Different Size Dust Distributions. Fluid-Part. Sep. J..

[58] Jaroszczyk T., Wake J., Connor M.J. (1993). Factors Affecting the Performance of Engine Air Filters. J. Eng. Gas Turbines Power.

[59] Jaroszczyk T. Air Filtration in Heavy-Duty Motor Vehicle Applications. Proceedings of the Dust Symposium III.

[60] Walsh D., Stenhouse J. (1997). The effect of particle size, charge, and composition on the loading characteristics of an electrically active fibrous filter material. J. Aerosol Sci..

[61] Zhang W., Deng S., Wang Y., Lin Z. (2020). Modeling the surface filtration pressure drop of PTFE HEPA filter media for low load applications. Build. Environ..

[62] Poon W.S., Liu B.Y.H. (1997). Dust Loading Behavior of Engine and General Purpose Air Cleaning Filters.

[63] Lee J.-K., Kim S.-C., Liu B.Y.H. (2001). Effect of Bi-Modal Aerosol Mass Loading on the Pressure Drop for Gas Cleaning Industrial Filters. Aerosol Sci. Technol..

[64] Saleh A., Tafreshi H.V. (2014). A simple semi-numerical model for designing pleated air filters under dust loading. Sep. Purif. Technol..

[65] Saleh A., Fotovati S., Tafreshi H.V., Pourdeyhimi B. (2014). Modeling service life of pleated filters exposed to poly-dispersed aerosols. Powder Technol..

[66] Dziubak T. (2013). Experimental research of axial cyclones of combustion engines air filters. Bull. Mil. Univ. Technol..

[67] Dziubak T. (2001). The dust sucking-off from the air filter multicyclone of the vehicle engine exploited in high pollution concentration conditions. Zagadnienia Eksploat. Masz. PAN.

[68] Cheng Y.H., Tsai C.J. (1998). Factors Influencing Pressure Drop Through a Dust Cake During Filtration. Aerosol Sci. Technol..

[69] Thomas D., Penicot P., Contal P., Leclerc D., Vendel J. (2001). Clogging of fibrous filters by solid aerosol particles Experimental and modelling study. Chem. Eng. Sci..

[70] Fotovati S., Tafreshi H.V., Pourdeyhimi B. (2012). A macroscale model for simulating pressure drop and collection efficiency of pleated filters over time. Sep. Purif. Technol..

